# Distinct amino acid motifs carrying multiple positive charges regulate membrane targeting of dysferlin and MG53

**DOI:** 10.1371/journal.pone.0202052

**Published:** 2018-08-09

**Authors:** Lu Zhou, Volker Middel, Markus Reischl, Uwe Strähle, G. Ulrich Nienhaus

**Affiliations:** 1 Institute of Applied Physics, Karlsruhe Institute of Technology (KIT), Karlsruhe, Germany; 2 Institute of Nanotechnology, Karlsruhe Institute of Technology (KIT), Karlsruhe, Germany; 3 Institute of Toxicology and Genetics, Karlsruhe Institute of Technology (KIT), Karlsruhe, Germany; 4 Institute for Applied Computer Science, Karlsruhe Institute of Technology (KIT), Karlsruhe, Germany; 5 Department of Physics, University of Illinois at Urbana-Champaign, Urbana, Illinois, United States of America; Consejo Superior de Investigaciones Cientificas, SPAIN

## Abstract

Dysferlin (Dysf) and mitsugumin53 (MG53) are two key proteins involved in membrane repair of muscle cells which are efficiently recruited to the sarcolemma upon lesioning. Plasma membrane localization and recruitment of a Dysf fragment to membrane lesions in zebrafish myofibers relies on the presence of a short, polybasic amino acid motif, WRRFK. Here we show that the positive charges carried by this motif are responsible for this function. In mouse MG53, we have identified a similar motif with multiple basic residues, WKKMFR. A single amino acid replacement, K279A, leads to severe aggregation of MG53 in inclusion bodies in HeLa cells. This result is due to the loss of positive charge, as shown by studying the effects of other neutral amino acids at position 279. Consequently, our data suggest that positively charged amino acid stretches play an essential role in the localization and function of Dysf and MG53.

## Introduction

Even under normal physiological levels of mechanical stress, skeletal muscle cells continuously suffer from plasma membrane lesions. A sophisticated machinery exists that swiftly closes these lesions with a repair patch consisting of proteins and lipids [1,2] so as to avoid cell death and unnecessary cycles of muscle regeneration resulting in the depletion of stem cell pools. In this work, we focus on two proteins, Dysf and MG53, that play essential roles in the membrane repair process that restores cell integrity [3,4]. Dysf is a type II membrane protein containing seven C2 domains and a membrane-spanning α-helix. In humans, mutations in the *DYSF* gene give rise to limb-girdle muscular dystrophy type 2B (LGMD2B), Miyoshi myopathy or distal myopathy with anterior tibialis onset [5,6]. It was shown earlier that Dysf binds phosphatidylserine (PS) in a Ca^2+^-dependent manner through its C2 domains [7]. Dysf can be cleaved by Ca^2+^-dependent calpain proteases; the product, mini-Dysf, consists only of the last two C2 domains and the membrane-spanning α-helix. This truncated Dysf isoform was shown to be actively involved in membrane repair in mammals [8,9]. In zebrafish, an even shorter isoform containing only the transmembrane helix was still able to accumulate at the site of lesion and rescue patch formation in Dysf mutants and morphants [9,10]. Recently, we identified a motif rich in basic amino acids (AAs), WRRFK, in zebrafish Dysf that is required for Dysf-mediated PS accumulation at the repair patch [11]. Multiple sequence alignment shows that this motif is conserved among a variety of species (S1 Fig), which suggests that it has a particular functional significance.

To fulfill its function in membrane repair, Dysf interacts with various other proteins. Among these, annexins form multimeric complexes in a Ca^2+^-dependent manner at the site of the lesion [10,12–14]. Muscle-specific protein caveolin-3 (Cav3) also associates with Dysf and regulates its membrane localization and endocytosis [15,16]. Moreover, MG53, a tripartite motif protein (TRIM72), is another component of the membrane repair machinery. A complex formed by Dysf, Cav3 and MG53 was suggested to be essential for membrane repair [3,17].

MG53 is rapidly recruited to the damage site in a process involving oxidation-dependent oligomerization [3]. Similar to Dysf, MG53 interacts with PS and mediates vesicle accumulation at the damage site [3]. In contrast to Dysf, however, accumulation of MG53 during the membrane repair process is not Ca^2+^- but Zn^2+^-dependent [3,18]. MG53 deletion results in muscle pathology with defective repair of the damaged membrane [3,17]. In addition to its role in membrane repair, MG53 regulates membrane budding and exocytosis [19]. MG53 consists of two domains, TRIM and SPRY, which are both required for proper subcellular localization of MG53 [19].

In murine MG53, we found a polybasic AA motif, WKKMFR, similar to WRRFK in zebrafish Dysf. It is also conserved among a variety of species but not among different TRIM proteins of the same species (*Mus musculus*), as shown by multiple sequence alignment (S2 Fig). These findings suggest a particular functional role for this motif in MG53. The motif is located at the very beginning of the N-terminal PRY extension of the SPRY domain [20] and, thus, between the TRIM and SPRY domains and may play a role as a targeting signal. Here we show that its modification can severely affect the proper membrane localization of MG53. In both Dysf and MG53, positive charges in their polybasic motifs play essential roles in their membrane targeting and function.

## Materials and methods

### Sample preparation and plasmids

In this work, we have used the zebrafish AB2O2 WT line (European Zebrafish Resource Centre (EZRC), KIT, Karlsruhe, Germany). Zebrafish husbandry and experimental procedures were performed in accordance with European and German animal protection regulations. According to Article 1 (3) of the EU Directive 2010/63/EU and the national legislation (§§ 7–9 of the Animal Welfare Act TierSchG and §14 of the German national regulation, TierSchVersV), this work does not involve animal experiments that need to be regulated by authorities since all animals used were larvae before the stage of self-feeding. Accordingly, we used replacement methods of animal experiments. Animal husbandry was approved by the responsible local authorities, the regional council Regierungspräsidium Karlsruhe, and is regularly checked by the regional veterinary office. Husbandry was approved with AZ 35–9185.64/BH KIT by the regional council. For anesthesia, embryos were submerged in fish water containing 0.018% tricaine at room temperature during imaging. Euthanasia of adults was performed by hypothermic shock (rapid chilling) for at least 20 min followed by decapitation.

For microinjection, embryos were injected (FemtoJet, Eppendorf, Leipzig, Germany) through the chorion directly into the yolk during the 1–2 cell stage. Purified plasmid DNA (20–80 ng/μl; midiprep, Qiagen, Hilden, Germany), diluted in distilled H_2_O and supplemented with 0.01% phenol red were used for injection. Injection needles were pulled from borosilicate glass capillary tubes with filament (0.58 mm diameter, Warner Instruments, Hamden, CT) using a micropipette puller (Sutter Instruments, Novato, CA). Injected embryos were grown to 3–5 dpf at 28°C before imaging. Zebrafish were immobilized on a microscope cover slide using 0.5% low melting point agarose supplemented with 0.02% ethyl 3-aminobenzoate methanesulfonate (MESAB, Sigma-Aldrich, St. Louis, MO).

HeLa cells were cultured in Dulbecco’s Modified Eagle’s Medium (DMEM) supplemented with 10% fetal bovine serum (FBS), 100 ng/mL streptomycin and 60 μg/mL penicillin (Thermo Fisher Scientific, Carlsbad, CA) at 37°C and 5% CO_2_. Before transfection, cells were seeded into an 8-well Lab-Tek II chambered coverglass (Thermo Fisher Scientific) and incubated for 24 h. Cells were transfected with Lipofectamine 3000 (Thermo Fischer Scientific) according to the manufacturer's protocol. After transfection, cells were incubated for 24 h and washed twice with DMEM containing 10% FBS before imaging. HeLa cells were always imaged at 37°C and 5% CO_2_.

The MG53:turboGFP plasmid was obtained from OriGene (OriGene, Rockville, MD). We noticed that it had a purely cytosolic distribution and did not target the plasma membrane. This observation agrees with earlier work reporting that MG53:GFP is mislocalized in the cytosol, whereas the inverse fusion GFP:MG53 localized correctly at the cell membrane [19]. Therefore, we cloned turboGFP:MG53 on the basis of the MG53:turboGFP plasmid. MG53 was amplified by PCR using forward and reverse primers with *XhoI* and *XbaI* restriction enzyme sites, respectively. An empty pcDNA3.1(+) vector (Thermo Fisher Scientific) was cut with the same restriction enzymes, and the *XhoI*-MG53-*XbaI* was inserted. For subcloning of turboGFP, primers with *KpnI* and *NotI* restriction sites were used during PCR. A linker with the sequence GSAGSSAAGSGEF [21] was used to concatenate turboGFP and MG53. It was inserted into the pcDNA3.1(+) vector containing turboGFP-MG53 after cutting with *XhoI*. The Dysf-Clover plasmids for zebrafish WRRFK-TM-C and human WRRFR-TM-C used in this study were described in reference [11]. Constructs were modified by site-directed mutagenesis using PCR. mRFP-Rab5 and mRFP-Rab7 were gifts from Ari Helenius (Addgene plasmid #14436); mRFP-ubiquitin was a gift from Nico Dantuma (Addgene plasmid #11935).

### Live confocal microscopy

Zebrafish were imaged on a Leica TCS SP2 confocal microscope with a water dip-in objective (HCX APO water; 63×/0.90, Leica Microsystems, Mannheim, Germany) at room temperature. Zebrafish myofibers were damaged via two-photon absorption using a Ti:Sa laser (Mai Tai, Spectra Physics, Mountain View, CA) set to 860 nm. To quantify protein accumulation after membrane damage, we measured the fluorescence intensity at the lesion in 9–15 independent experiments, using ImageJ for analysis. The significance was assessed by a two-sided Welch’s t-test with Bonferroni correction or Student’s t-test coded in Matlab.

HeLa cells were investigated with an Andor Revolution XD spinning disk confocal laser scanning microscope (BFi OPTiLAS, München, Germany) equipped with an Apo N 60×/1.49 oil immersion objective (Olympus, Hamburg, Germany). The fluorescence of LysoTracker Blue DND-22, turboGFP/Clover and mRFP was excited with 405 nm, 488 nm and 561 nm lasers, the emission was filtered with 447/60 (peak wavelength/width), 525/50 and 607/50 band pass filters (Semrock, New York, NY), respectively. Membranes were locally damaged by using the FRAPPA unit of the microscope for irradiating a region of 6 × 6 pixels (~1.3 × 1.3 μm^2^) with 405 nm laser light (200 mW at the specimen). The selected area was scanned with 400 μs pixel dwell time; scanning was repeated 400×. After membrane damage, image sequences were acquired with 3 s intervals. The fluorescence intensity at the lesion site was analyzed with ImageJ.

## Results

### A positively charged motif affects Dysf accumulation in the repair patch and its plasma membrane localization

**Fig 1:**
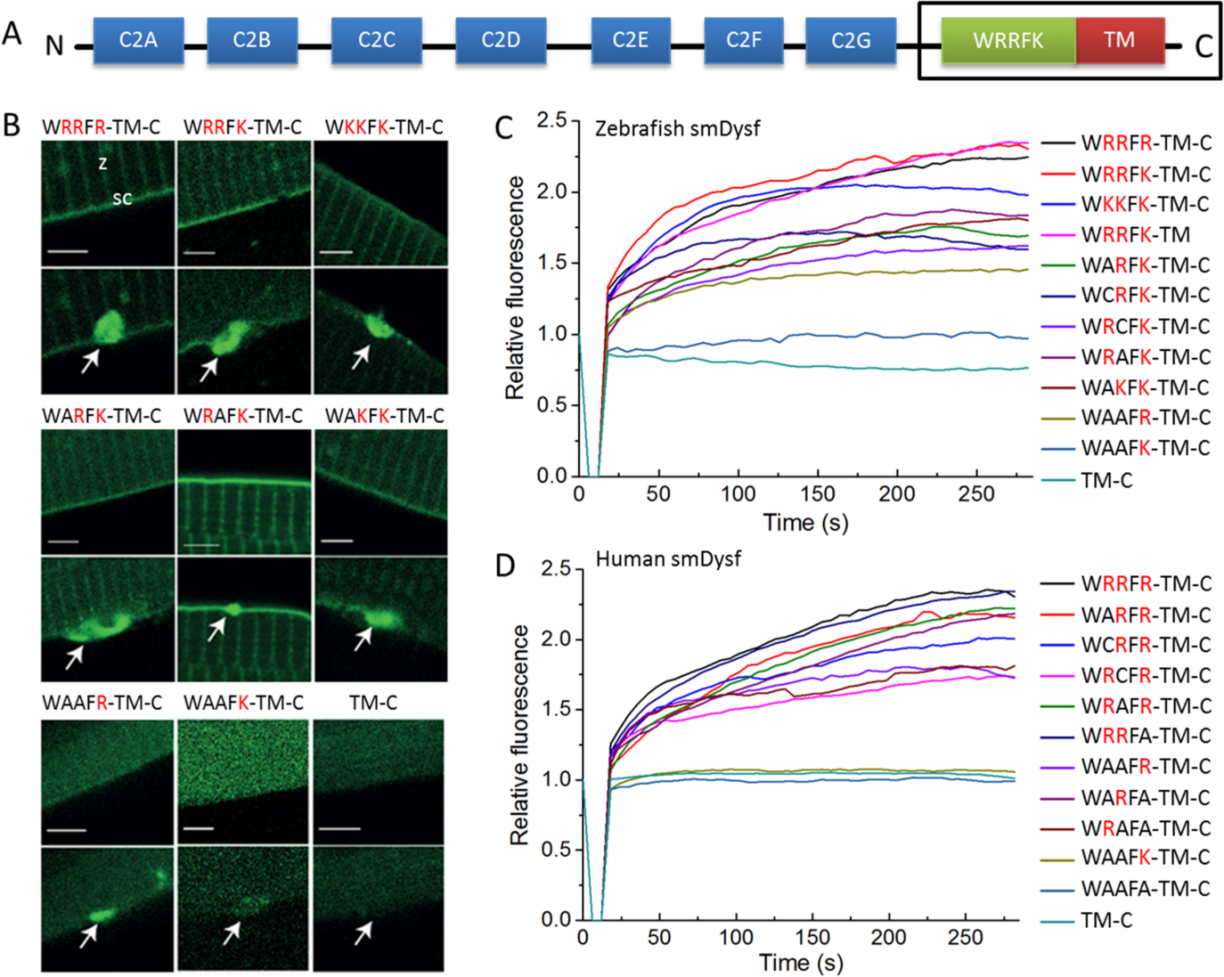
Accumulation of smDysf at the lesion site depends on the positive charge of the WRRFK motif *in vivo*. (A) Domain structure of Dysf. The boxed fragment is smDysf, which was used to test accumulation at the lesion patch. (B) Representative images showing accumulation of smDysf and different mutants. Basic AAs are highlighted in red; arrows indicate the site of lesion. Z-line (z) and sarcolemmal (sc) regions are noted. (C-D) Corresponding kinetics of accumulation of zebrafish (C) or human (D) smDysf at the damage site, again with the basic AAs highlighted in red. The fluorescence intensity at the lesion was normalized to the one of the undamaged state. Intensity courses are averages over 9–15 damaged cells. Scale bars, 4 μm.

We have previously shown that an N-terminal fragment of the zebrafish Dysf protein (super-mini Dysf, smDysf) spanning the WRRFK motif and the TM domain (Fig 1A) suffices for its accumulation in the repair patch [11]. Moreover, this protein fragment rescues transport of PS to the repair patch in Dysf deficient animals [11]. A key physical property of this motif is the presence of positive charges on the three basic residues. We thus asked what happens to the accumulation of smDysf at the lesion site upon membrane injury if those residues are modified and, specifically, if the overall charge is reduced. To this end, we generated nine different variants by replacing the arginines and/or lysine with lysines, arginines and/or alanines and tested them in the skeletal musculature of zebrafish embryos. Zebrafish were always injected with the same amount of plasmid, and only well-expressing myofibers with bright fluorescence were selected for membrane damage analysis (Fig 1B). Substituting arginines by lysines or vice versa did not lead to a significant change of accumulation (Fig 1B and 1C), suggesting that it is not so much the AA structure but the positive charge that matters. In mutants WARFK-TM-C, WRAFK-TM-C, and WAKFK-TM-C, the net positive charge was reduced, resulting in impaired accumulation at the site of lesion (Fig 1B and 1C). Further replacement of two arginines to alanines in WAAFR-TM-C and WAAFK-TM-C led to even poorer accumulation. Complete removal of the WRRFK motif abolished the recruitment of the fragment to the lesion site (Fig 1B and 1C; TM-C). The same phenotypes were observed with wild type and mutants of the human Dysf fragment (WRRFR-TM-C) when tested in zebrafish (Fig 1D). A significance analysis of the data is shown in S3 Fig. The two tryptophan residues on the two sides of the charged motif (WRRFKW) were also tested. Both ARRFKW-TM-C and ARRFKA-TM-C showed comparable accumulation with the wildtype control smDysf after membrane damage, which indicates that they did not affect recruitment of smDysf (S4 Fig).

**Fig 2:**
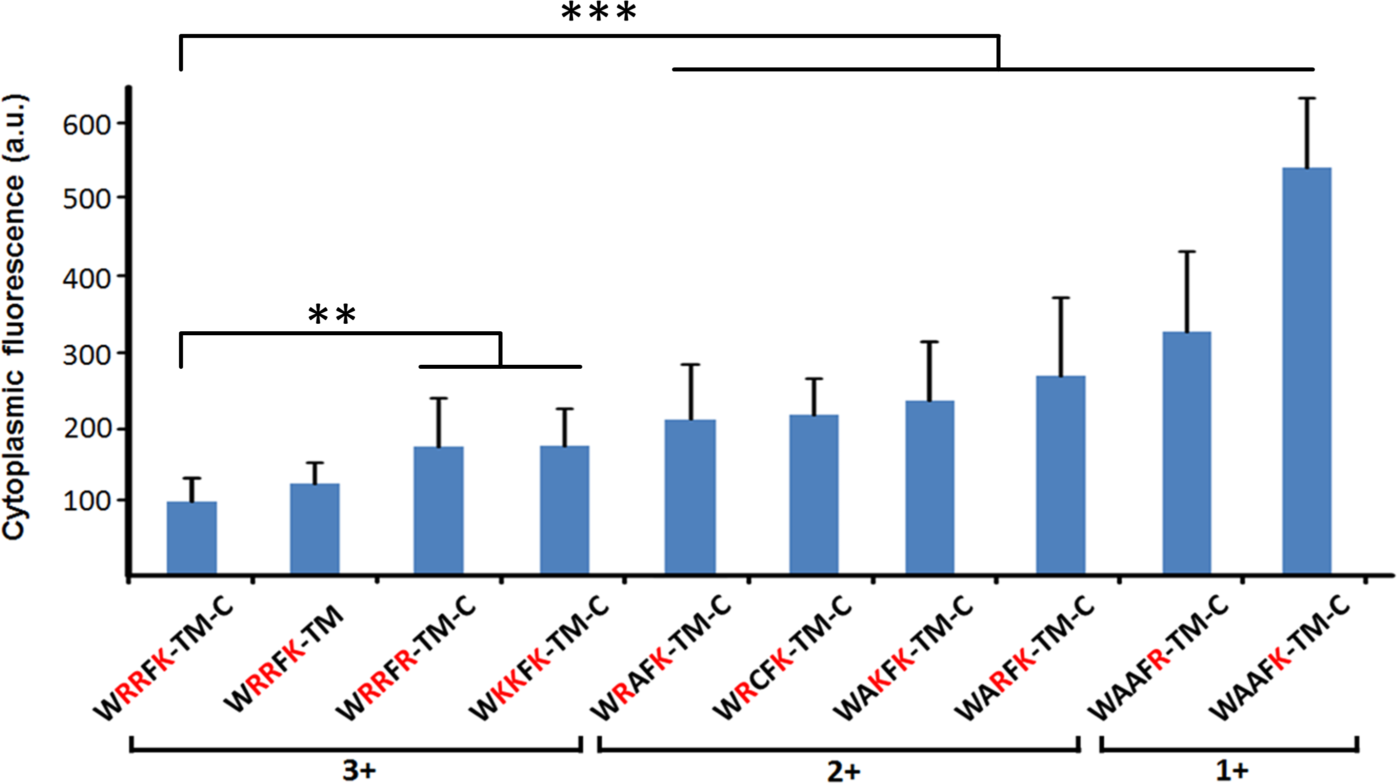
The cellular distribution of smDysf depends on the positive charge of the WRRFK motif. The cytoplasmic fraction is significantly increased upon reducing the net charge but not by polypeptide truncation on the C-terminal side of smDysf. For this plot, the cytoplasmic fluorescence of 9–15 myofibers was measured and averaged for each mutant. All data are referenced to the wildtype set to 100% and plotted as mean ± SD. The original data are provides as S1 Dataset. Zebrafish embryos expressing these ten variants were injected, treated and imaged under identical conditions. The mutant sequences are grouped according to the net charge (1+– 3+) of the different motifs under physiological conditions. Significance was tested against the wildtype control smDysf (WRRFK-TM-C) by Student’s t-test (** *p* < 0.01, *** *p* < 0.001).

We quantitatively analyzed the cytoplasmic fluorescence of zebrafish embryos with different smDysf mutants. All myofibers (9–15 for each mutant) tested in Fig 1 were used to calculate the mean cytoplasmic fluorescence intensity before membrane damage. Interestingly, the positive charge also determined the localization of the Dysf fragments. Constructs with a decreased positive charge showed an enhanced cytoplasmic distribution and, consequently, a reduced plasma membrane and T-tubule localization in the absence of injury (Fig 2). Thus, the increased accumulation in the repair patch of positively charged protein fragments may be a simple consequence of their greater abundance in the plasma membrane prior to lesioning.

### Mutation K279A in the WKKMFR motif leads to MG53 mislocalization

**Fig 3:**
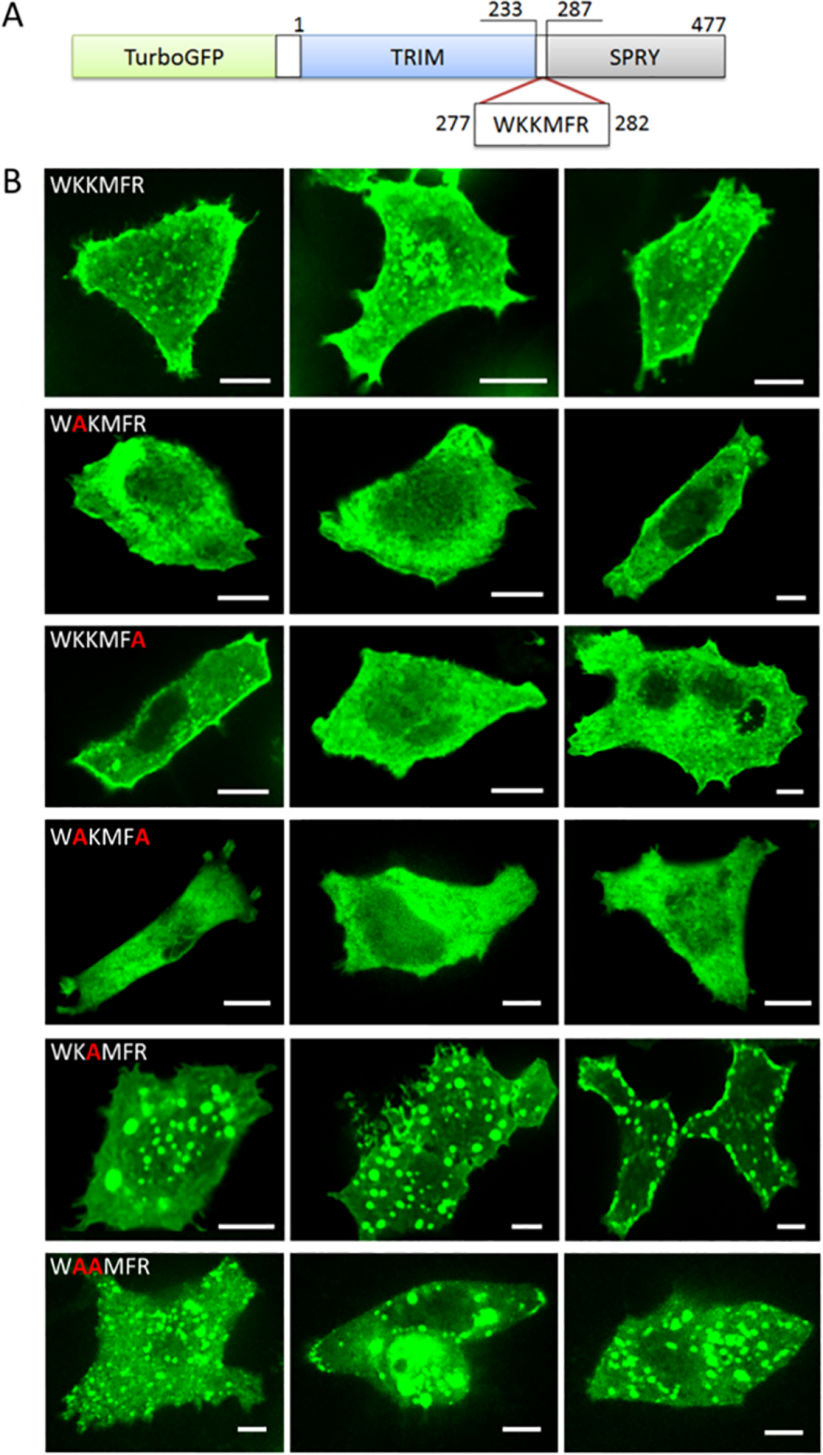
Mutation K279A leads to mislocalization of MG53. (A) Domain structure of the fusion protein turboGFP:MG53. (B) Cellular localization of (top to bottom) turboGFP:MG53 and mutants K278A, R282A, K278A/R282A, K279A, K278A/K279A in HeLa cells. Three representative images are shown for each variant, all imaged with excitation at 488 nm. Scale bars, 10 μm.

**Fig 4:**
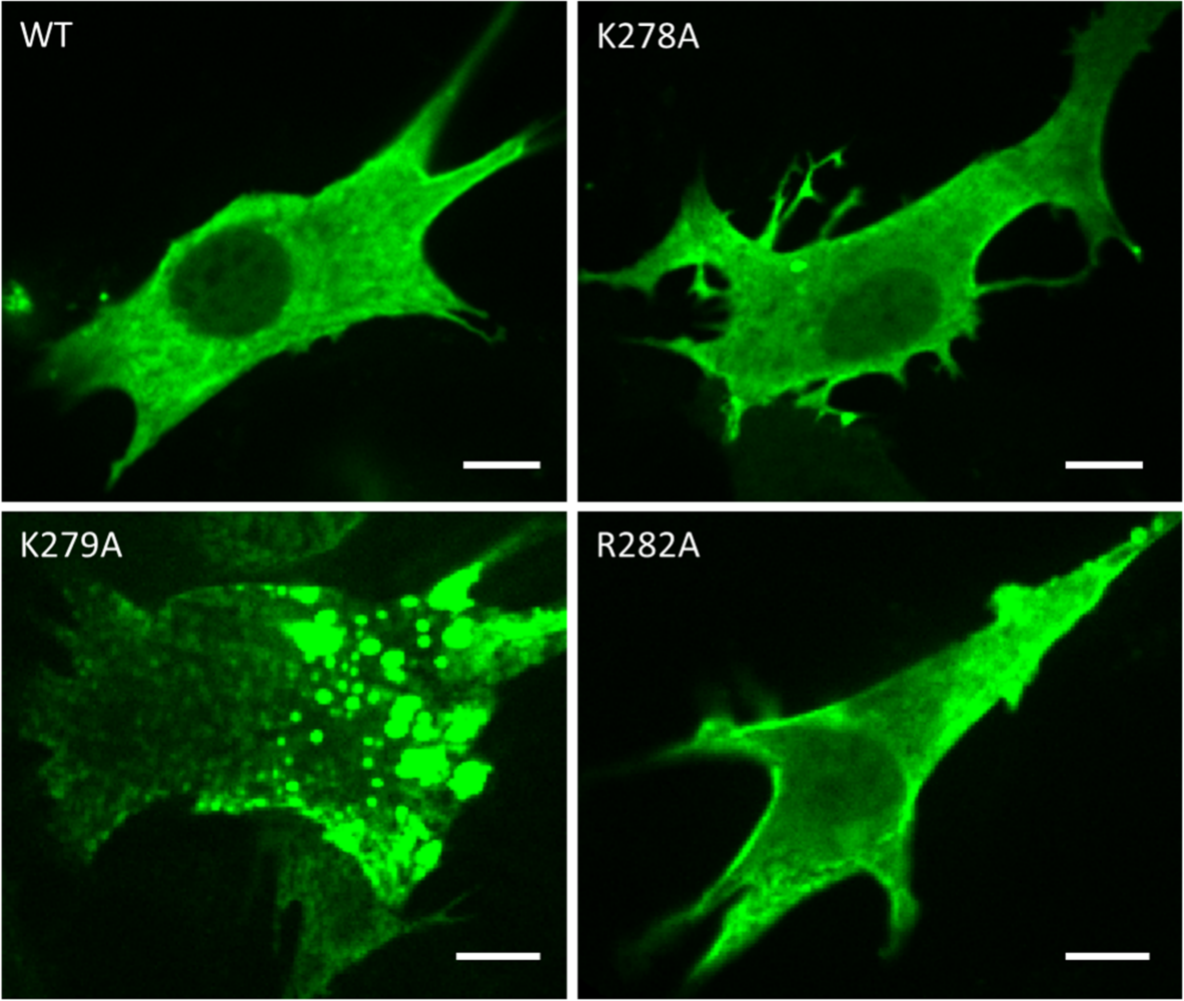
Confocal images of C2C12 myoblasts expressing turboGFP:MG53 and mutants K278A, R282A, K279A. Fluorescence was excited with a 488-nm laser. As in HeLa cells, K279A shows substantial vesicular localization in myoblasts. Scale bars, 10 μm.

In mouse MG53, we found a 6-AA motif, WKKMFR, at residue positions 277–282, which also contains three positively charged residues and resembles the WRRFK motif in zebrafish Dysf. According to Ref. [3], this motif is located in the linker region (residues 233–287) between the TRIM and SPRY domain. Of note, in other papers, the separation between TRIM and SPRY is shown between residues 284 and 285, without any linker [19,22]. In the x-ray structure, the motif is found at the very beginning of the N-terminal PRY extension of the SPRY domain (Fig 3A) [20]. We studied the fusion protein turboGFP:MG53 and its mutants K278A, K279A and R282A in transfected HeLa cells to examine the relevance of the basic AAs. Similar to turboGFP:MG53, the single mutants K278A and R282A distributed both on the membrane and in the cytosol, whereas the loss of two positive charges in the combined variant K278A/R282A resulted in a failure to target the plasma membrane. Surprisingly, K279A showed a very unusual phenotype with a lot of very bright speckles, indicating that the protein aggregated in intracellular vesicles. The same observation was made with the double mutant K278A/K279A (Fig 3B). As a result, there was much less membrane targeting than in the wild type (Fig 3B). For single-point mutations in the motif, the identical behavior was observed in C2C12 myoblasts (Fig 4). A similar vesicular distribution was also observed with isolated TRIM domains (residues 1–284) by Cai et al. [19]. These results suggest that the second lysine, K279, in the 6-AA motif in a linker region plays an important role for the proper localization of MG53.

### MG53(K279A) is sequestered in inclusion bodies

**Fig 5:**
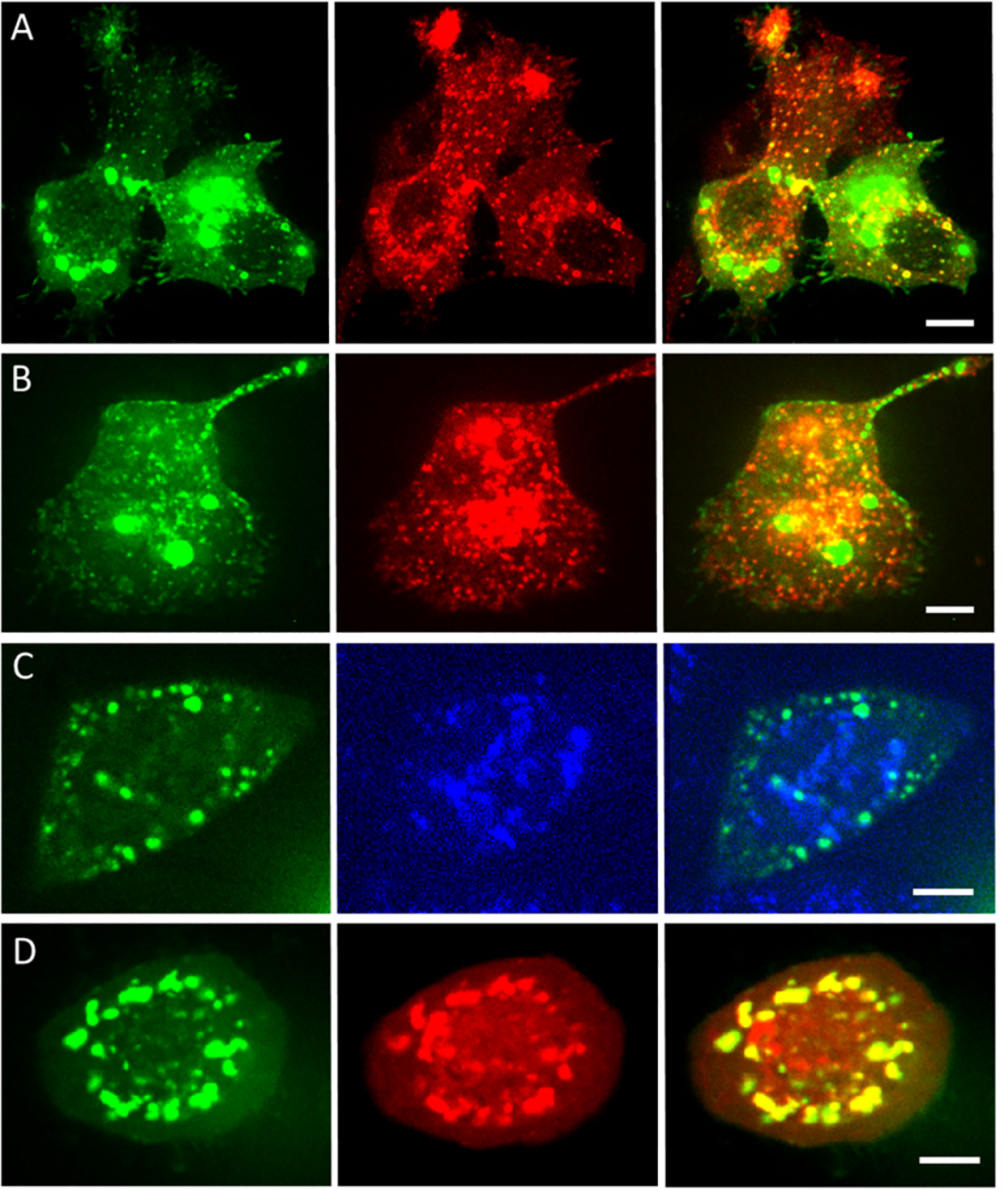
Colocalization analysis by dual-color confocal imaging. Two-channel imaging of turboGFP:MG53 (green fluorescence excited with 488-nm light, first column) with (second column) early endosomes, excited with 561-nm light (A), late endosomes, excited with 561-nm light (B), lysosomes, excited with 405-nm light (C), and inclusion bodies, excited with 561-nm light (D). Overlay images are shown in the third column. Scale bars, 10 μm.

We imaged HeLa cells in two color channels to check colocalization of the MG53(K279A)-enriched vesicles with early endosomes, late endosomes, lysosomes and inclusion bodies. Early and late endosomes were labeled with mRFP-Rab5A and mRFP-Rab7 [23], respectively. We found distinct colocalization of small green vesicles containing turboGFP:MG53(K279A) with red early and late endosomes (Fig 5A and 5B). For the big bright MG53(K279A)-enriched vesicles, however, there was no colocalization with early or late endosomes. We also did not find colocalization with lysosomes, labeled with LysoTracker Blue DND-22 (Thermo Fisher Scientific, Carlsbad, CA) according to the manufacturer’s protocol (Fig 5C). Finally, we labeled inclusion bodies with mRFP-ubiquitin [24] to check their colocalization with MG53(K279A). Surprisingly, the MG53(K279A)-enriched vesicles showed a high degree of colocalization with inclusion bodies (Fig 5D). Inclusion bodies are generally formed upon aggregation of proteins, which will be degraded through autophagy [25]. The single mutation K279A thus results in the formation of MG53 protein aggregates sequestered in inclusion bodies.

### Mislocalization of MG53(K279A) results from a loss of positive charge

**Fig 6:**
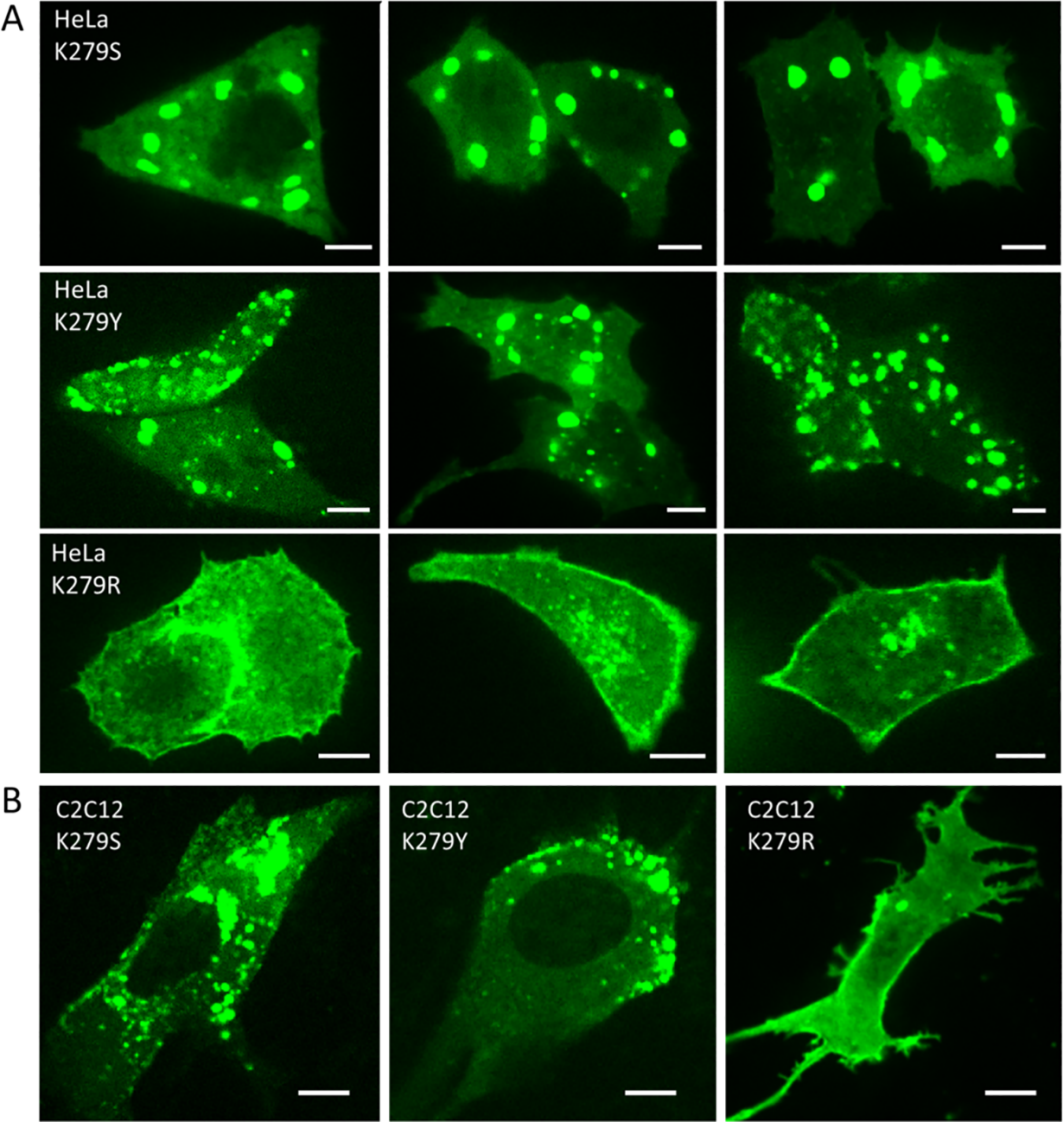
Cellular localization of turboGFP:MG53 mutants K279S, K279Y and K279R in HeLa cells and C2C12 myoblasts. Representative images of (A) HeLa cells and(B) C2C12 myoblasts show that the charge-maintaining modification K279R rescues membrane targeting of MG53. Fluorescence was excited with a 488-nm laser. Scale bars, 10 μm.

Mutation of K279 to alanine leads to severe mislocalization. To obtain more insight into the mechanism, we mutated K279 to serine (K279S) and tyrosine (K279Y). However, those variants also showed severe aggregation and lacked membrane targeting (Fig 6A). Then we further enquired whether the missing positive charge is the reason for this phenotype. Therefore, we replaced K279 by arginine to retain a positive charge at that position. Indeed, MG53(K279R) showed correct membrane and cytoplasmic distribution as the wildtype in HeLa cells; no aggregation was observed (Fig 6A). Identical results were found in C2C12 myoblasts (Fig 6B). These results indicate that aggregation of MG53(K279A) occurs upon a loss of positive charge at this position, which is consistent with the observations made with smDysf.

## Discussion

Lesions in the cell membrane lead to rapid recruitment of repair proteins such as Dysf and MG53 to the damage site, where they contribute to formation of repair patches that temporarily close the wound to prevent cell death. The C-terminal short fragments of both zebrafish (WRRFK-TM-C) and human (WRRFR-TM-C) Dysf (smDysf) are swiftly recruited to the lesion site after membrane damage [11]. Their efficient accumulation requires positive charges from arginine and lysine residues in the WRRFK or WRRFR motifs; removal of basic residues or the entire 5-AA motif leads to significant deficiencies in the repair function of smDysf. Our present work shows that cellular localization of smDysf depends on the positive charges; reducing their number results in an enhanced cytoplasmic distribution (Fig 2). Therefore, efficient targeting of smDysf to the plasma membrane relies on the presence of positively charged AAs in the 5-AA motif, which presumably is a prerequisite for the membrane repair function of Dysf.

Unlike Dysf, the repair protein MG53 does not have a transmembrane domain. Nevertheless, it is capable of targeting the plasma membrane and accumulating at membrane lesions [3]. Sequence analysis revealed a positively-charged 6-AA motif in mouse MG53, WKKMFR, that is similar to the WRRFK and WRRFR motifs in zebrafish and human Dysf. Because such motifs are also present in MG53 of other organisms including human (WRKMFR), monkey (WRKMFR), frog (WRKMFR), panda (WRKMFR), chameleon (WRKMYR), we pursued a mutational study to see whether the motif is essential for MG53 functioning in membrane repair. We found that, different from Dysf, a single lysine mutated to alanine (K279A) leads to a complete loss of plasma membrane localization. Instead, the protein accumulates in large vesicles in both HeLa cells and C2C12 myoblasts. Co-localization studies showed that MG53(K279A) ends up in inclusion bodies, indicating severe protein aggregation. Therefore, we conclude that K279 in the linker region between the TRIM and SPRY domains plays a crucial role for the correct localization of MG53. It is likely important for its membrane repair function but perhaps also for its role in ubiquitination of the insulin receptor [26].

Interestingly, Cai et al. [19] reported that removal of the entire SPRY domain (from M285 on) led to a similar vesicular distribution as in K279A. This result suggests that the WKKMFR motif must reside in the linker region between the TRIM and SPRY domains to properly perform its function. Presumably, if the motif is only attached to the TRIM domain as a C-terminal tail, K279 and/or the other basic residues may form salt bridges with acidic residues on the domain surface, making it inaccessible for interactions that promote correct localization and function.

Two possible reasons for MG53(K279A) mislocalization could be (1) loss of a positive charge at a critical position as in smDysf and/or (2) failure of modifying K279, e.g., through phosphorylation. With the MG53(K279R) variant, we showed that the positively charged, basic arginine instead of lysine maintains correct membrane targeting. We examined two other AA residues for replacement of K279, tyrosine and serine, which are polar and neutral and are frequently phosphorylated. However, both variants did not show membrane localization, lending further support to the notion that proper membrane targeting relies on the positive charge at this position. Both Dysf and MG53 have been shown to interact in rat liver cells *in vitro* [27]. Therefore, it is tempting to speculate that they might form a complex together with phosphatidylserine (PS). Alternatively, the sole interaction of MG53 with PS could also mediate its localization to the plasma membrane [3,11,27]. Furthermore, it has been shown that MG53 binds to exposed cholesterol in complex with cavin-1 during membrane repair. This may be yet another way for MG53 to associate with the plasma membrane, at least during wound repair [27]. Altogether, comparison of the two basic motifs in Dysf and MG53 reveals an important role of the positive charges for plasma membrane localization and repair.

## Supporting information

S1 Dataset EXCEL spreadsheet containing the data set from which the averages and error bars in Fig 2 were calculated. (XLSX)

S1 Fig Multiple sequence alignment of the Dysf transmembrane domain, the polybasic WRRFK motif and flanking amino acids. The alignment shows conservation of the positive motif across various species. In some instances, the fourth and fifth AAs are replaced by similar ones (F/Y, K/R). The color code indicates identity of the shown amino acids. The percentages of coverage (cov) and identity (pid) are referenced to *Danio rerio*. Multiple alignment was done using MView 1.63.
(TIF)

S2 Fig Multiple sequence alignment of the polybasic WKKMFR motif of murine MG53 and flanking amino acids. (A) The alignment shows conservation of the motif across various species, only the second lysine is replaced by arginine in some species. (B) The motif is not conserved among different Trim proteins of the same species, however. The color code indicates identity of the shown amino acids. The percentages of coverage (cov) and identity (pid) are referenced to *Mus musculus*. Multiple alignment was done using MView 1.63.
(TIF)

S3 Fig Significance blots of zebrafish and human smDysf variants accumulation. Zebrafish (A) and human (B) smDysf variants were tested for significance against the wildtype control smDysf (zebrafish: WRRFK-TM-C; human: WRRFR-TM-C). For both zebrafish and human smDysf, reducing the net positive charge increase the difference between the control and variants. The two sided Welch’s t-test with Bonferroni correction was performed.
(TIF)

S4 Fig Tryptophans in the WRRFKW motif do not influence accumulation of zebrafish smDysf. (A) Kinetics of accumulation of zebrafish wildtype control smDysf (WRRFKW-TM-C, black) and tryptophan mutants ARRFKW-TM-C (red) and ARRFKA-TM-C (blue) at the damage site (mean ± SD). (B) Significance test shows these is no significant difference between the mutants and the control. The two-sided Welch’s t-test with Bonferroni correction was performed.
(TIF)
