# Supplementary figures and images for: Distinct amino acid motifs carrying multiple positive charges regulate membrane targeting of dysferlin and MG53
Source: PLoS One. 2018 Aug 9;13(8):e0202052. doi: 10.1371/journal.pone.0202052 (PMC6084962; doi:10.1371/journal.pone.0202052)

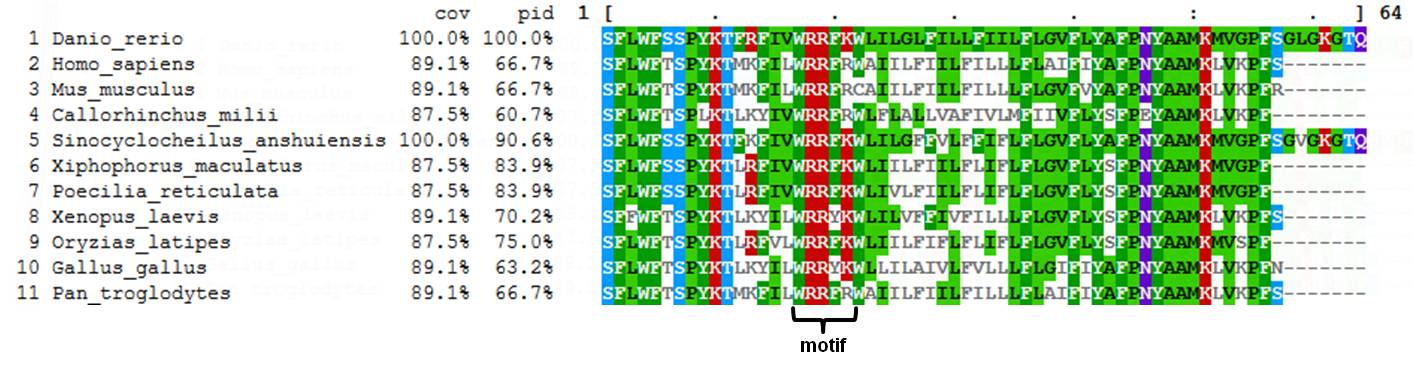

Supplement: S1 Fig — The alignment shows conservation of the positive motif across various species. In some instances, the fourth and fifth AAs are replaced by similar ones (F/Y, K/R). The color code indicates identity of the shown amino acids. The percentages of coverage (cov) and identity (pid) are referenced to Danio rerio. Multiple alignment was done using MView 1.63. (TIF) [file pone.0202052.s002.tif]

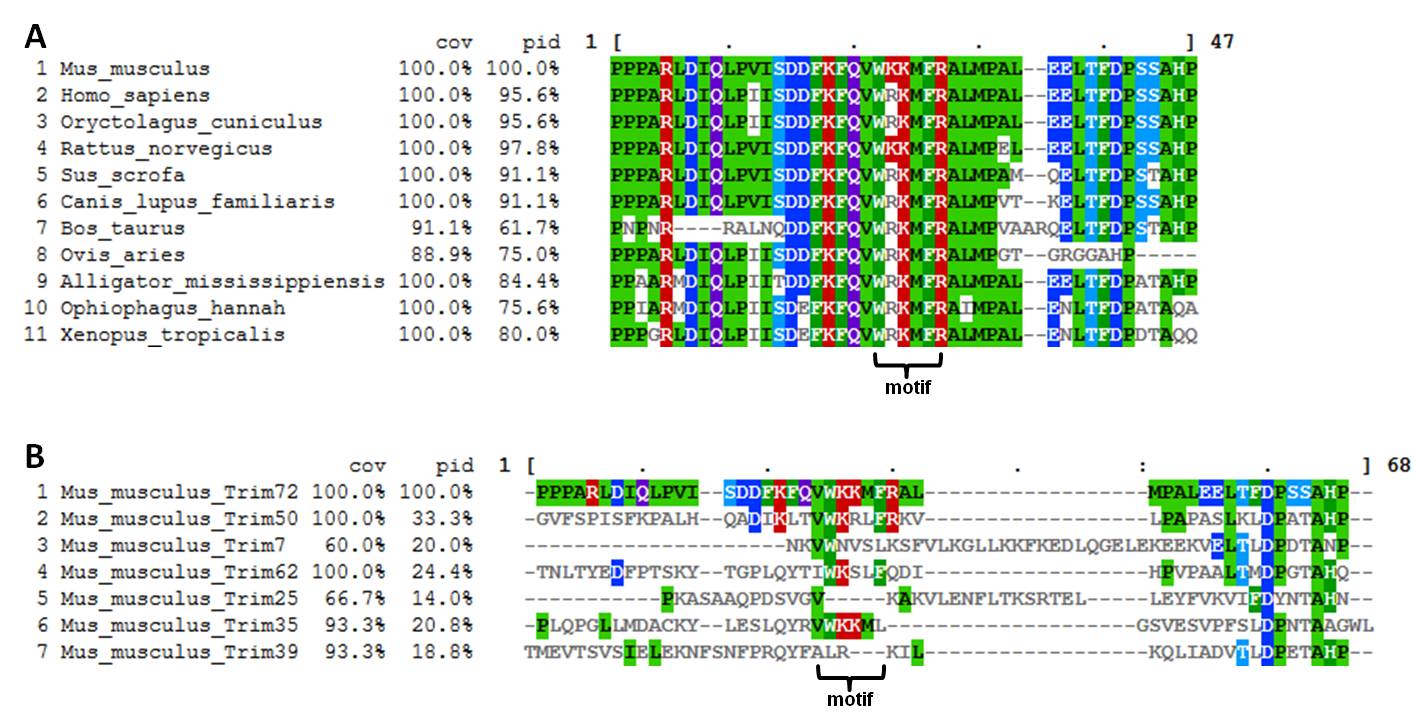

Supplement: S2 Fig — (A) The alignment shows conservation of the motif across various species, only the second lysine is replaced by arginine in some species. (B) The motif is not conserved among different Trim proteins of the same species, however. The color code indicates identity of the shown amino acids. The percentages of coverage (cov) and identity (pid) are referenced to Mus musculus. Multiple alignment was done using MView 1.63. (TIF) [file pone.0202052.s003.tif]

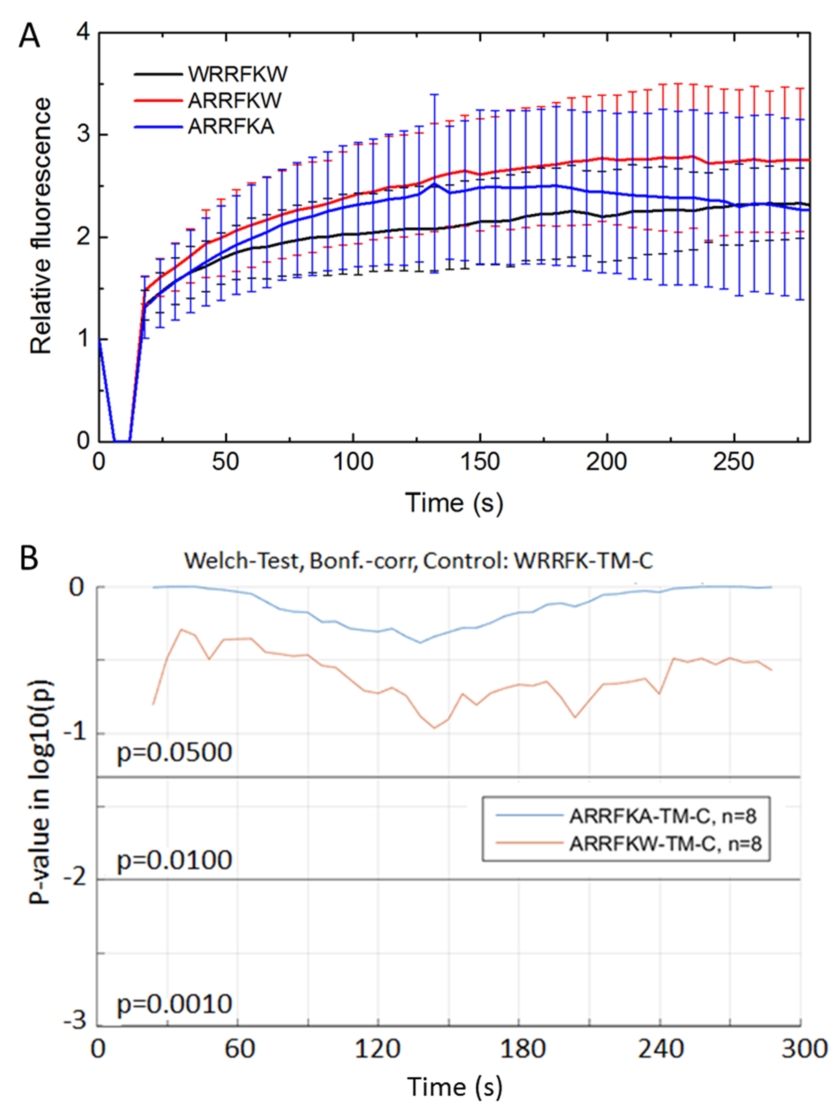

Supplement: S3 Fig — Zebrafish (A) and human (B) smDysf variants were tested for significance against the wildtype control smDysf (zebrafish: WRRFK-TM-C; human: WRRFR-TM-C). For both zebrafish and human smDysf, reducing the net positive charge increase the difference between the control and variants. The two sided Welch’s t-test with Bonferroni correction was performed. (TIF) [file pone.0202052.s004.tif]

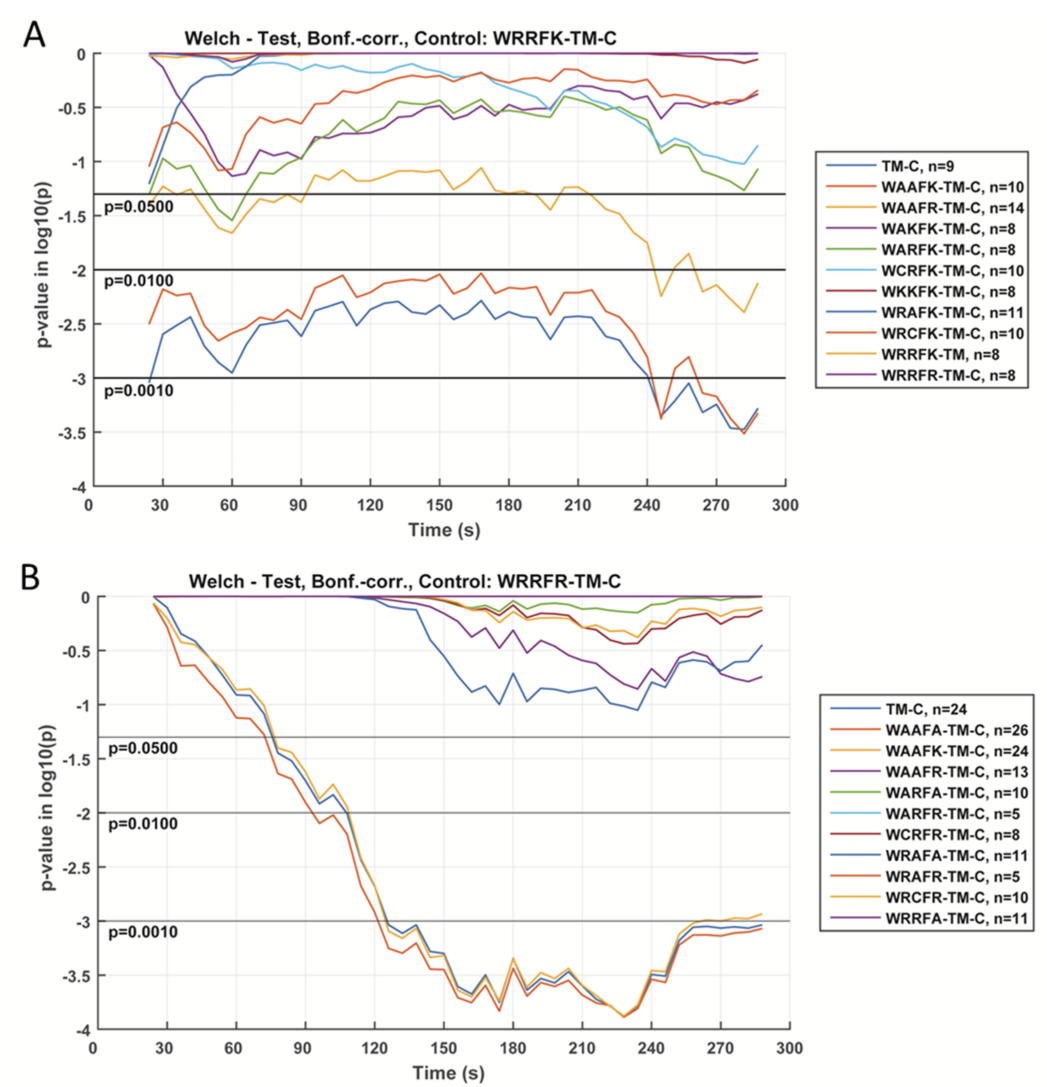

Supplement: S4 Fig — (A) Kinetics of accumulation of zebrafish wildtype control smDysf (WRRFKW-TM-C, black) and tryptophan mutants ARRFKW-TM-C (red) and ARRFKA-TM-C (blue) at the damage site (mean ± SD). (B) Significance test shows these is no significant difference between the mutants and the control. The two-sided Welch’s t-test with Bonferroni correction was performed. (TIF) [file pone.0202052.s005.tif]
